# ﻿*Peperomia kauaiana* (Piperaceae), a new alternate-leaved species from Kaua‘i, Hawaiian Islands and notes on two possibly extinct Hawaiian *Peperomia*

**DOI:** 10.3897/phytokeys.269.173971

**Published:** 2026-01-12

**Authors:** Kenneth R. Wood, Warren L. Wagner, Susan Fawcett

**Affiliations:** 1 National Tropical Botanical Garden, 3530 Papalina Road, Kalāheo, HI 96741, USA National Tropical Botanical Garden Kalāheo United States of America; 2 Department of Botany, Smithsonian Institution, PO Box 37012, Washington, DC 20013-7012, USA Department of Botany, Smithsonian Institution Washington United States of America; 3 University and Jepson Herbaria, 1001 Valley Life Sciences Building #2465, University of California, Berkeley, CA 94720-2465, USA University of California Berkeley United States of America

**Keywords:** ‘ala‘ala wai nui, endangered species, Hawaiian flora, pepper family, plant extinction prevention, single-island endemism, subgenus *Micropiper*

## Abstract

A new species of *Peperomia* with alternate leaves from Kaua‘i, Hawaiian Islands, is described and illustrated, with notes on its conservation status, distribution and ecology. We present a dichotomous key to all five Hawaiian *Peperomia* species with alternate leaves and include notes on two possibly extinct Hawaiian *Peperomia* species, namely *P. degeneri* and *P. subpetiolata*. *Peperomia kauaiana***sp. nov.** differs morphologically from its Hawaiian congeners by its unique combination of diminutive leaves 5–14(–18) mm long, 4–11(–14) mm wide, palmately 5- to 7-nerved, ovate to ovate-orbicular with margins revolute, petioles 2–5 mm long and spikes 11–17(–22) mm long. Plants have been documented in three distinct windward Kaua‘i locations to date, including the southern ridges of Wahiawa, the central ridges of Wai‘ahi and the north-eastern ridges of the Makaleha Mountains. *Peperomia kauaiana* represents a newly-described wet forest species endemic to the island of Kaua‘i and is currently in need of conservation. Its discovery raises the total number of endemic Hawaiian *Peperomia* species to 24 and single-island endemic *Peperomia* on Kaua‘i to three.

## ﻿Introduction

*Peperomia* Ruiz & Pav. (Piperaceae) is a pantropical genus that contains ca. 1426 accepted species (POWO 2025). The Hawaiian Islands have the greatest number of *Peperomia* taxa in the Pacific with 23 endemic and two indigenous. *Peperomia* is also one of the few Hawaiian genera with native species arising from a number of separate colonisation events (Wagner et al. 1990, 1999; Lim et al. 2019). Specifically, phylogenetic analyses indicate that the Hawaiian taxa are the result of four *Peperomia* lineages that dispersed from the Neotropics, including two separate colonisations of indigenous taxa and two other colonisations that gave rise to the Hawaiian endemic radiations (Lim et al. 2019). The endemic Hawaiian species are currently placed into the subgenus Micropiper (Miq.) Miq., distinguished in having fruit uniformly covered with sticky papillae (Frenzke et al. 2015, 2016). The viscid fruit easily adheres to whatever it is in contact with, a trait that is most likely a key factor for long distance dispersal by birds to remote oceanic islands (Yuncker 1933; Baldwin and Wagner 2010; Frenzke et al. 2016).

Most Hawaiian *Peperomia* are multi-island species, although nine are single-island endemics with three on Maui, two each on Kaua‘i and Moloka‘i and one each on Hawai‘i (i.e. Big Island) and O‘ahu. Botanists at the National Tropical Botanical Garden (NTBG), in concert with other government and non-government agencies, have spent decades exploring and documenting *Peperomia* throughout the Hawaiian and South Pacific islands, thereby further expanding phytogeographical and phylogenetic knowledge of *Peperomia* (Wagner et al. 1990, 1999; Wood 2012; Lim et al. 2019; Lorence and Wagner 2020). In 1991, during botanical surveys around the Wahiawa Mountains of southern Kaua‘i, Tim Flynn (curator of the PTBG Herbarium) documented an unusual, diminutive, alternate-leaved species of *Peperomia*. Several years later (i.e. 1993), he found another colony of this undescribed taxon in the north-eastern mountains of Makaleha, Kaua‘i. In his collection notes, Flynn described the unusual habitat to be “along spine of ridge in moss layer under uluhe (*Dicranopteris linearis* (Brum.f.) Underw.)”. Subsequently, in 2019 and 2020, additional colonies were documented around the central windward ridges of eastern Kaua‘i, confirming Flynn’s collections to be highly distinctive from all other alternate-leaved species and we hereby present its formal description.

## ﻿Material and methods

All collection sites were accessed by helicopter transport. Botanical voucher collections of *Peperomia kauaiana* are curated at the PTBG Herbarium, with duplicates distributed at BISH, CAS, MO, NY and US. All photo images were made by the authors unless otherwise noted. All morphological measurements were taken from dried herbarium specimens and field notes and are presented in the descriptions as follows: length × width, followed by units of measurements (mm, cm). The inflorescence of *Peperomia* is referred to as a spike throughout this manuscript and its measurements include both the length of peduncle and fertile rachis. We assessed the extinction risk for *P. kauaiana* using the IUCN Red List Categories and Criteria (IUCN 2012, 2024) and follow Wood et al. (2019) for citing *Peperomia* extinctions in the Hawaiian Islands. The extent of occurrence (EOO) and area of occupancy (AOO) were calculated by using ArcMap 10.6.1 (ESRI 2018) in relation to coordinates recorded while collecting herbarium specimens or making field observations. Geographic coordinates have been truncated to protect exact locations from unauthorised access.

## ﻿Taxonomic treatment

### ﻿ Peperomia kauaiana

Taxon classificationPlantaePiperalesPiperaceae

K.R.Wood & W.L.Wagner sp. nov.

6C345A09-A9E1-5307-8B1B-4D7AEC5D749B

urn:lsid:ipni.org:names:77374762-1

Figs 1, 2, 3A–C, 4

#### Diagnosis.

*Peperomia kauaiana* is morphologically most similar to *P. degeneri* Yunck. from which it differs by the following combination of characteristics: stem internodes 3–5 mm long (vs. 10–15 mm long), leaves 5- to 7-nerved, ovate to ovate-orbicular, 0.5–1.4(–1.8) cm long, margins revolute (vs. leaves 3-nerved, elliptic to oblong-elliptic, 1.5–2(–3.5) cm long, margins flat), petioles 0.2–0.5 cm long (vs. petioles 0.8–1.2 cm long) and spikes 1.1–1.7(–2.2) cm long (vs. spikes 2.5–4.8 cm long).

**Figure 1.**
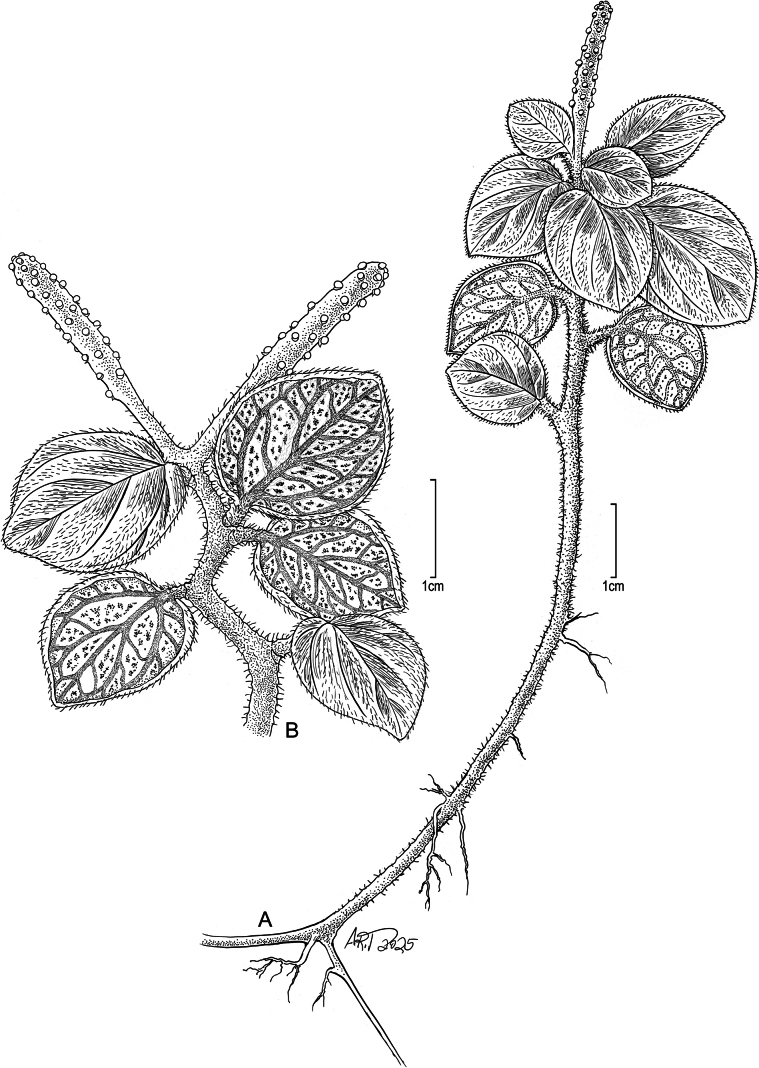
*Peperomia kauaiana***A.** Plant with lower stem rooting at nodes; **B.** Upper portion of plant with short spikes and detail of leaf venation pattern and pubescence. Drawn from: **A***Wood 18149* (isotype, US) **B** field photos, Wai‘ahi, 22 Oct 2020, *Wood et al. 18580*. Illustration by Alice Tangerini.

**Figure 2.**
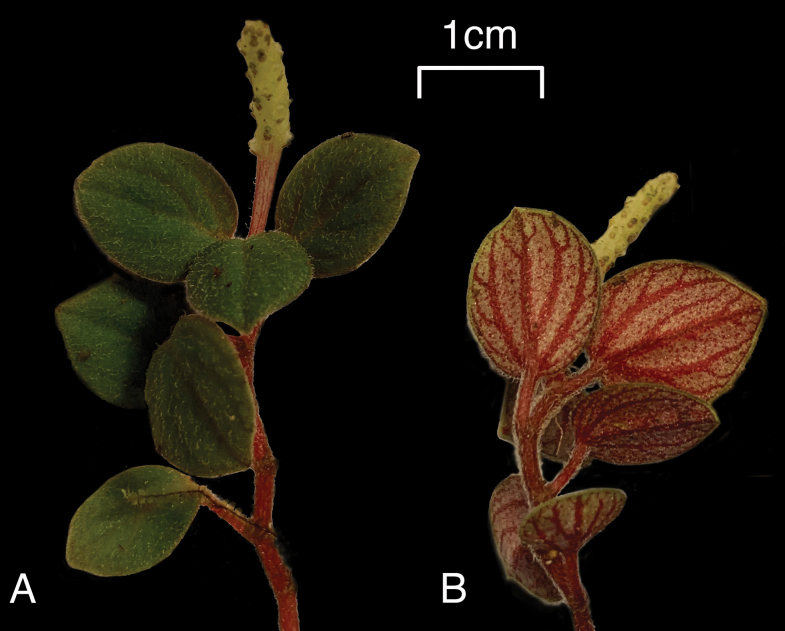
*Peperomia kauaiana***A.** Fertile plant showing pubescent adaxial leaf surface and terminal spike; **B.** Same plant showing pubescent abaxial leaf surface with distinctive primary and secondary venation and alternate revolute leaves. Field photos: **A, B** Wai‘ahi, 22 Oct 2020, *Wood et al.18580*.

**Figure 3.**
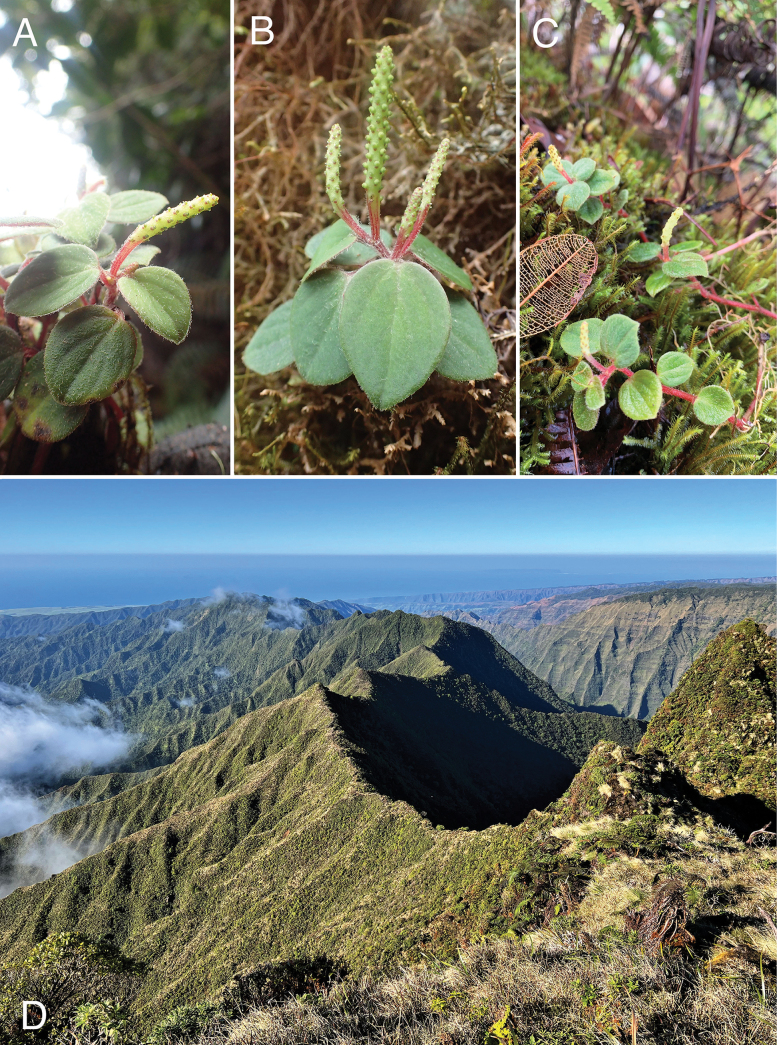
**A**–**C.***Peperomia kauaiana*, fertile plants *in situ*, decumbent to sub-erect habit with terminal spikes, terrestrial in moss; **D.** View from Kawaikini looking south along the windward ridgeline of eastern Kaua‘i showing buttressed ridges descending to the east (left), which are prime habitat for *Peperomia kauaiana*. Field photos: **A, B** Wai‘ahi, 4 Apr 2019, *Wood et al. 18149***C** Wai‘ahi, 22 Oct 2020, *Wood et al. 18580***D** photo taken 28 Jan 2022.

**Figure 4.**
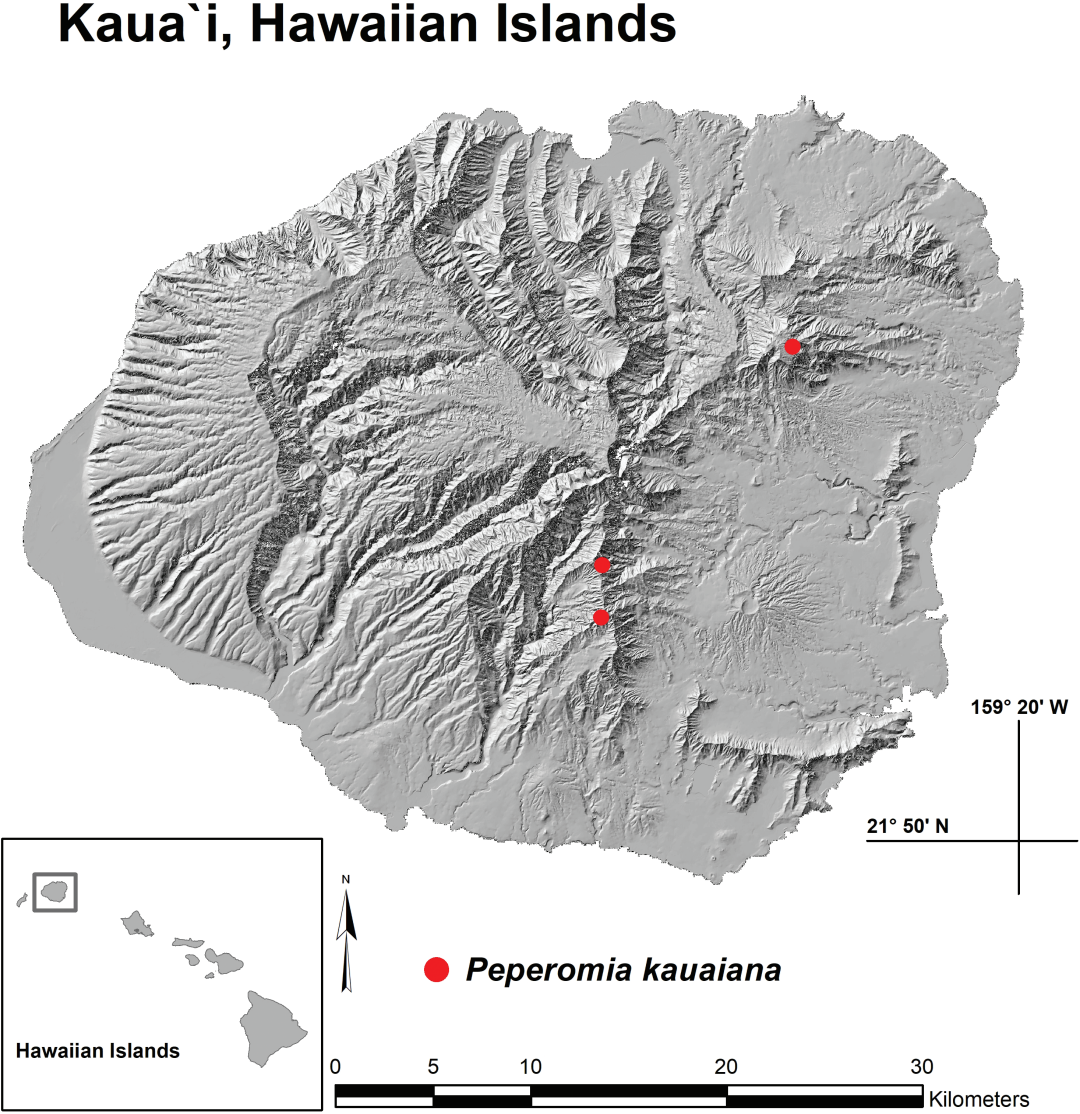
Distribution map with red dots indicating the three known locations of *Peperomia kauaiana*, Kaua‘i, Hawaiian Islands. Upper right dot is the Makaleha site, middle dot is the Wai‘ahi site and lower dot is the Wahiawa site.

#### Type.

**USA. Hawaiian Islands, Kaua‘i**: • Līhu‘e District, Wai‘ahi, 22.014, -159.501, 830 m alt., 4 Apr 2019 (fl. & fr.), *Wood, Perlman & Query 18149* (holotype: PTBG 1000067990!; isotypes: BISH!, CAS!, MO!, NY!, US!).

#### Description.

***Herb***, terrestrial, stems dark red to red-purple, decumbent or occasionally sub-erect, rooting at nodes, unbranched, rarely 2- to 3-branched in the upper part, 3–8 cm long, 1–2 mm in diameter towards the base; internodes 3–5 mm long, densely appressed hirtellous to strigulose, the hairs 0.3–0.5 mm long. ***Leaves*** alternate, thinly firm and fleshy, drying chartaceous, ovate, ovate-orbicular, rarely elliptic-ovate, 5–14(–18) mm × 4–11(–14) mm, palmately 5- to 7-nerved, upper surface dull, dark green, moderately appressed hirtellous, lower surface with veins usually dark red to purple, intercostal areas pale pink to red-purple or rarely green, moderately appressed hirtellous, margins revolute, apex acute, base subcordate to rounded or obtuse, rarely cuneate, petioles 2–5 mm long, densely appressed hirtellous. ***Spikes*** 1, rarely 2, terminal, 11–17(–22) mm long, peduncles red to purple, 4–7 × 0.3–0.4 mm, moderately appressed hirtellous, sometimes densely towards base, the fertile rachis glabrous, yellow-green, 7–14 × 1 mm, flowers moderately congested, bracts orbicular, ca. 0.5 mm diameter, stamens ellipsoidal, 2, ovary ovoid, apex oblique; stigmas terminal, shallowly cleft to bi-lobed. ***Fruits*** ovoid, 0.3–0.5(–0.8) mm long, dark brown, covered with sticky papillae.

#### Additional specimens examined

**(paratypes). USA. Hawaiian Islands, Kaua‘i: Kōloa District**, • **Wahiawa**, 670–975 m alt., 13 Apr 1991 (fr.), *Flynn et al. 4595* (BISH, PTBG, US) • **Kawaihau District, Makaleha Mountains**, 823–920 m alt., 2 Jul 1993 (fl. & fr.), *Flynn et al. 5398* (PTBG) • **Līhu‘e District, Wai‘ahi**, 847 m alt., 22 Oct 2020 (fl. & fr.), *Wood et al. 18580* (BISH, CAS, PTBG, US).

#### Phenology.

*Peperomia kauaiana* has been observed with flower and fruit during April, July and October.

#### Etymology.

The epithet refers to the island of Kaua‘i, oldest and most floristically rich of all the high Hawaiian Islands and the only known location for *Peperomia kauaiana*.

#### Vernacular name.

‘Ala‘ala wai nui is the Hawaiian name for related species. Hawaiians used the ash of their burned leaves and stems as a grey-green dye in kapa making (Krauss 2001).

#### Affinities.

Lim et al. (2019) presented a phylogenetic analysis of plastomes that indicate four major lineages of *Peperomia* within the Hawaiian archipelago. Although many relationships within these clades are poorly resolved, additional preliminary analyses, based on recently developed target-enrichment nuclear data using the *Angiosperms353* probe set, corresponds closely with the findings of Lim et al. (2019). The four colonisation events of *Peperomia* to the Hawaiian Islands represent the largest number of colonisation events of any flowering plant genus to the archipelago (Howarth et al. 1997; Keeley and Funk 2011; Lim et al. 2019). Based on morphological similarity, *Peperomia kauaiana* belongs to the largest of these radiations, designated as ‘Hawaiian radiation A’ by Lim et al. (2019).

**Figure 5.**
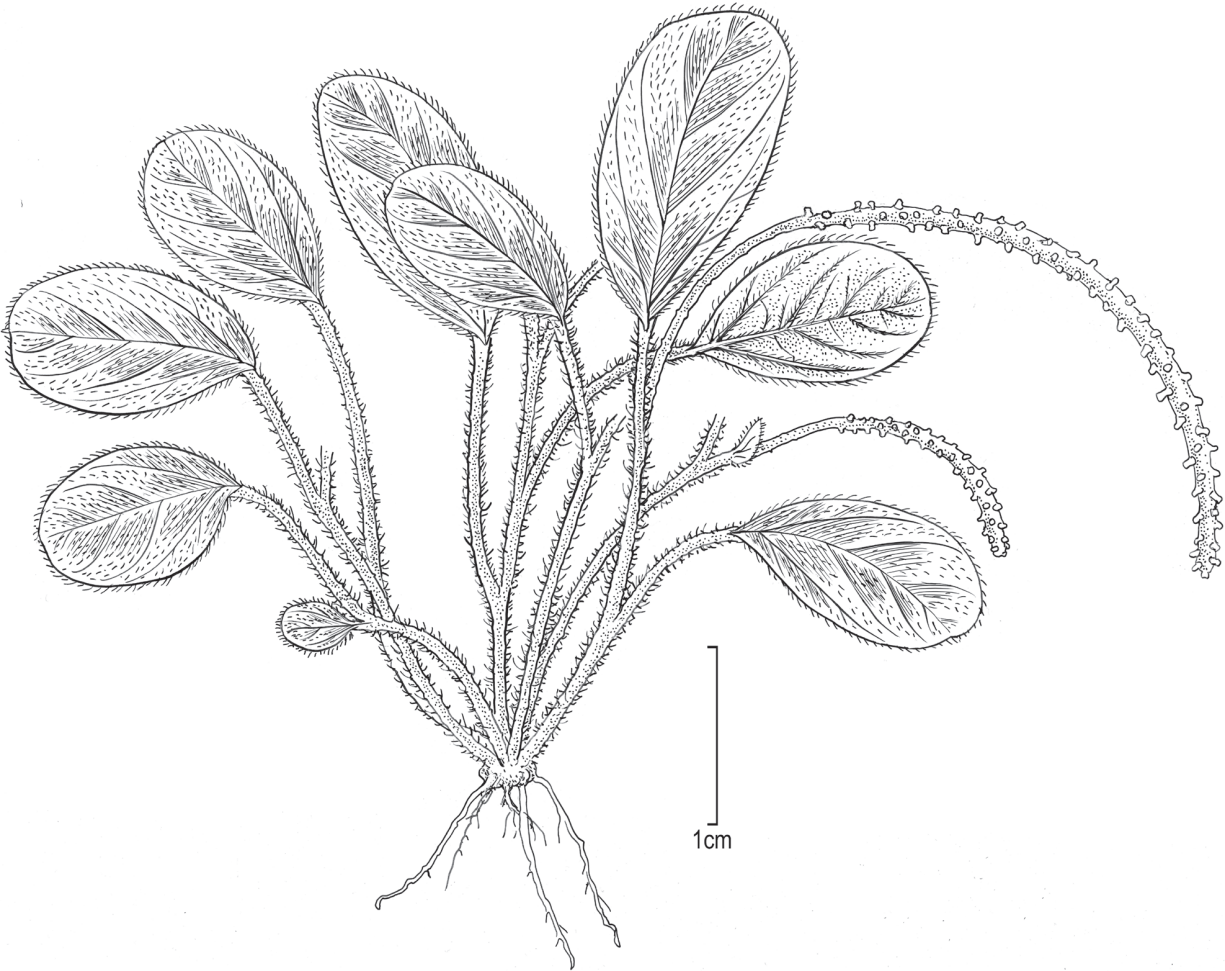
*Peperomia degeneri* Yunck. Habit showing stems, leaves, spikes and pubescence drawn from: Moloka‘i, Kalua‘aha Valley, 12 Jul 1928, O. *Degeneri & H. Wiebke 3061* (isotype, US). Illustration by Alice Tangerini.

**Figure 6.**
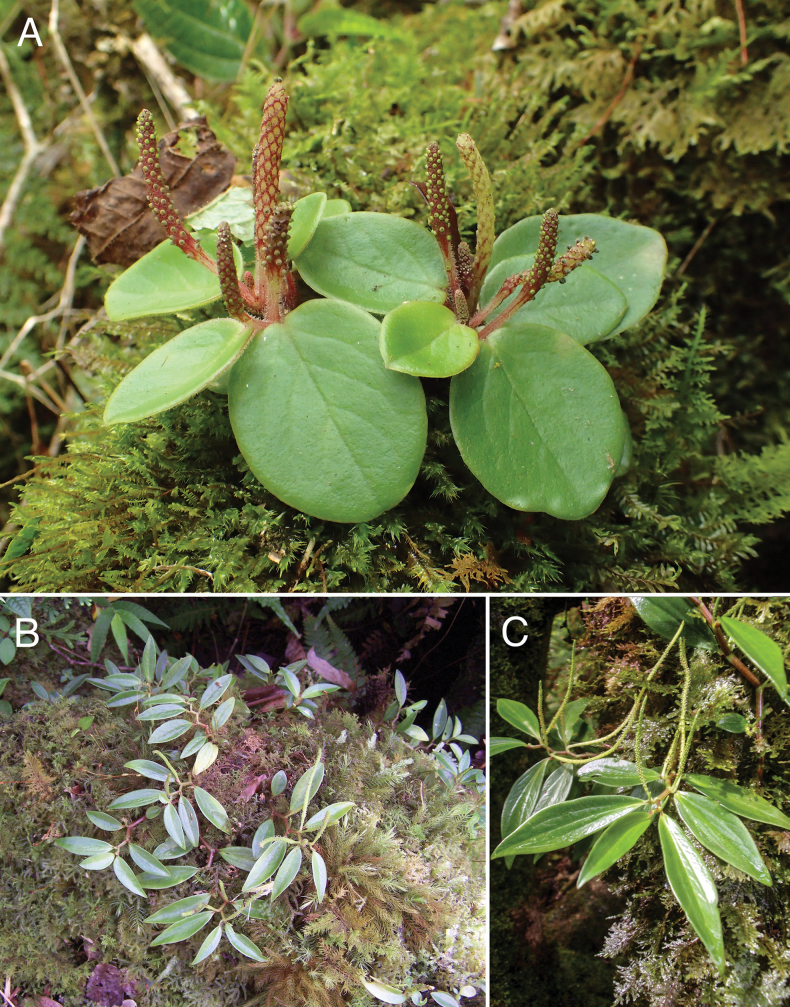
**A.***Peperomialatifolia, in situ*, showing glabrous adaxial leaves with immature and mature pikes; **B.***Peperomia alternifolia, in situ*, fertile on moss-covered log; **C.***Peperomiaoahuensis, in situ*, fertile and epiphytic on moss covered trunk. Field photos: **A** 17 May 2017, Lumaha‘i Valley, Kaua‘i, *Wood et al. 17396* (PTBG, US), **B** 24 Jan 2007, Honolua Valley, Maui, by Hank Oppenheimer, **C** 18 Jun 2008, Wainiha Valley, Kaua‘i, *Wood et al. 13117* (MBK, PTBG, UC).

Morphologically, *Peperomia kauaiana* is most similar to *P. degeneri* (Moloka‘i, Fig. 5), with both species having very small, strictly alternate leaves that are hirsute to densely hirtellous on both sides, yet *P. kauaiana* is generally a smaller plant and has revolute rather than flat leaf margins, along with other distinguishing characters noted in the diagnosis. Superficially, *P. kauaiana* can be confused with *P. cookiana* C.DC. (Kaua‘i, O‘ahu, Moloka‘i, Lāna‘i, Maui and Big Island of Hawai‘i), as both are relatively small herbs and have hirsute to hirtellous leaves and internodes. However, *P. kauaiana* has alternate leaves and *P. cookiana* has opposite or whorled leaves, 2–4 per node. Although *P. alternifolia* Yunck. (Moloka‘i, Lāna‘i and Maui, Fig. 6B) and *P. oahuensis* C.DC. (Kaua‘i and O‘ahu, Fig. 6C) are alternate-leaved like *P. kauaiana*, they differ in being entirely glabrous, whereas *P. kauaiana* has hirsute to hirtellous leaves, petioles, internodes and peduncles. The only other species that could potentially be confused with *P. kauaiana* is *P. latifolia* Miq. (Kaua‘i, O‘ahu, Moloka‘i, Lāna‘i, Maui and Big Island of Hawai‘i, Fig. 6A), yet *P. kauaiana* differs from *P. latifolia* in having stem internodes 3–5 mm long, densely appressed hirtellous to strigulose (vs. internodes 10–30(–80) mm long, hirsute to subglabrate), leaves strictly alternate 5–14(–18) mm × 4–11(–14) mm, with revolute margins (vs. leaves alternate, opposite or whorled (20–)30–60(–75) mm × (15–)20–50(–65) mm, margins flat), adaxial leaf surface appressed hirtellous (vs. adaxial leaf surface glabrous or hirsute only towards base), petioles 2–5 mm long (vs. petioles 7–20(–30) mm long) and spikes 11–17(–22) mm long, fertile rachis 1 mm wide (vs. spikes (15–)20–80(–150) m long, fertile rachis 2–3 mm wide) (see Table 1 and key to Hawaiian *Peperomia* with alternate leaves). Note: We examined type collections of *P. dentulibractea* Miq. (Moloka‘i), *P. punaluuna* C.DC. (O‘ahu), *P. villipeduncula* C.DC. (O‘ahu) and *P. waihoiana* St.John (Maui), that were later synonymised with *P. latifolia* by Yuncker and others (de Condolle 1913; Yuncker 1933; Wagner et al. 1990, 1999) and agree that they fall within the circumscription of *P. latifolia*.

**Table 1.** Comparison of morphological characters for all five alternate-leaved Hawaiian *Peperomia* species.

Species	*P. alternifolia*	*P. degeneri*	*P. kauaiana*	*P. latifolia*	*P. oahuensis*
**Plant length**	(5–)10–15(–30) cm	5 cm	3–8 cm	1.5–8(–20) cm	5–30 cm
**Internode length**	3–10(–15) mm	10–15 mm	3–5 mm	10–30(–80) mm	4–60 mm
**Internode pubescence**	glabrous	hirsute	densely appressed hirtellous	hirsute to subglabrate	glabrous
**Leaf arrangement**	alternate	alternate	alternate	Lower part alternate or opposite upper part opposite or whorled	alternate
**Leaf shape**	elliptic-oblanceolate or rhombic-elliptic	elliptic to oblong-elliptic, rarely spatulate	ovate, ovate-orbicular, rarely elliptic-ovate	elliptic-obovate to subrhombic-obovate, broadly elliptic, or orbicular, sometimes subspatulate	elliptic-lanceolate, sometimes lanceolate, or rarely narrowly subrhombic
**Leaf length**	1.5–4 cm	1.5–2(–3.5) cm	0.5–1.4(–1.8) cm	(2–)3–6(–7.5) cm	4.5–10(–12) cm
**Leaf width**	0.5–1 cm	0.8–1.5(–1.7) cm	0.4–1.1(–1.4) cm	(1.5–)2–5(–6.5) cm	1.4–2.5(–3) cm
**Leaf margins**	flat	flat	revolute	flat	flat
**Leaf pubescence abaxially**	glabrous	moderately to very sparsely hirsute	moderately appressed hirtellous	hirsute sometimes only along mid-rib or towards margins	glabrous
**Leaf pubescence adaxially**	glabrous	moderately to very sparsely hirsute	moderately appressed hirtellous	glabrous or hirsute towards base along mid-rib	glabrous
**Leaf venation**	3-nerved	3-nerved	5- to 7-nerved	3- to 5-nerved	3- to 5-nerved
**Petiole length**	0.3–0.6 cm	0.8–1.2 cm	0.2–0.5 cm	0.7–2(–3) cm	0.3–1 cm
**Spike length**	1.5–2.5(–3) cm	2.5–4.8 cm	1.1–1.7(–2.2) cm	(1.5–)2–8(–15) cm	4–7 cm
**Rachis width**	0.5–1 mm	1–1.5 mm	1 mm	2–3 mm	1.5–2 mm

#### Distribution and ecology.

*Peperomia kauaiana* is endemic to the volcanic island of Kaua‘i where it is has been documented in lowland wet forest around the island’s eastern windward slopes between 670 and 975 m elevation. Location sites include the south-eastern ridges of Wahiawa, the central eastern ridges of Wai‘ahi and the north-eastern ridges of the Makaleha Mountains (Fig. 4). It has predominantly been observed growing terrestrially in layers of bryophytes and in the understorey of ferns. These wet forests are low-statured, range between 3000 and 6500 mm of precipitation per year and are rich in endemic plant species. Dominant genera include trees of *Metrosideros* Banks ex Gaertn. (Myrtaceae) and *Cheirodendron* Nutt. ex Seem. (Araliaceae) and large patches of matting ferns, such as *Dicranopteris* Bernh. and *Diplopterygium* (Diels) Nakai (Gleicheniaceae). Other common tree and shrub genera include endemic species of *Polyscias* J.R.Forst. & G.Forst. (Araliaceae); *Pritchardia* Seem. & H.Wendl. (Arecaceae); *Dubautia* Gaudich. (Asteraceae); *Cyanea* Gaudich. (Campanulaceae); *Antidesma* L. (Phyllanthaceae); *Scaevola* L. (Goodeniaceae); *Hydrangea* Gronov. (Hydrangeaceae); *Geniostoma* (Loganiaceae); *Coprosma* J.R.Forst. & G.Forst., *Kadua* Cham. & Schltdl., *Psychotria* L. (Rubiaceae); and *Melicope* J.R.Forst. & G.Forst. (Rutaceae). Genera of sedges include *Carex* L., *Cyperus* L. and *Machaerina* Vahl (Cyperaceae). Smaller shrubs, herbs and vines include *Astelia* Banks & Sol. ex R.Br. (Asteliaceae); *Bidens* L. (Asteraceae); *Vaccinium* L. (Ericaceae); *Cyrtandra* J.R.Forst. & G.Forst. (Gesneriaceae); *Freycinetia* Gaudich. (Pandanaceae); and *Smilax* L. (Smilaceae). Occasional genera of ferns include *Sadleria* Kaulf. (Blechnaceae); *Cibotium* Kaulf. (Cibotiaceae); and *Microlepia* C.Presl (Dennstaedtiaceae). Other *Peperomia* species in the nearby vicinity of *P. kauaiana* include three predominantly terrestrial species, *P. cookiana*, *P. hesperomannii* Wawra and *P. latifolia* (Fig. 6A), along with one epiphytic species, *P. oahuensis* (Fig. 6C).

##### ﻿Key to Hawaiian *Peperomia* with alternate leaves

Note: H = Hawai‘i (Big Island); K = Kaua‘i; L = Lāna‘i; M = Maui; Mo = Moloka‘i; O = O‘ahu.

**Table:** 

1	Internodes hirsute	**2**
–	Internodes glabrous	**4**
2(1)	Leaves alternate, opposite or whorled, (2–)3–6(–7.5) cm long, (1.5–)2–5(–6.5) cm wide, spikes (1.5–)2–8(–15) cm long, rachis 2–3 mm wide; K, O, Mo, L, M, H	***P. latifolia***
–	Leaves strictly alternate, 0.5–2(–3.5) cm long, 0.4–1.5(–1.7) cm wide, spikes 1.1–4.8 cm long, rachis 1–1.5 mm wide	**3**
3(2)	Stem internodes 10–15 mm long, leaves 3-nerved, elliptic to oblong-elliptic, 1.5–2(–3.5) cm long, margins flat, petioles 0.8–1.2 cm long, spikes 2.5–4.8 cm long; Mo	***P. degeneri***
–	Stem internodes 3–5 mm long, leaves 5- to 7-nerved, ovate to ovate-orbicular, 0.5–1.4(–1.8) cm long, margins revolute, petioles 0.2–0.5 cm long, spikes 1.1–1.7(–2.2) cm long; K	***P. kauaiana***
4(1)	Leaves elliptic-oblanceolate or rhombic-elliptic, 1.5–4 cm long, 0.5–1 cm wide; spikes 1.5–2.5(–3) cm long; rachis 0.5–1 mm in diameter; Mo, L, M	***P. alternifolia***
–	Leaves elliptic-lanceolate, sometimes lanceolate, or rarely narrowly sub-rhombic, 4.5–10(–12) cm long, 1.4–2.5(–3) cm wide; spikes 4–7 cm long; rachis 1.5–2 mm in diameter; K, O	***P. oahuensis***

##### ﻿Preliminary conservation assessment. IUCN Red List Category.

When evaluated using the World Conservation Union (IUCN) criteria for endangerment (IUCN 2012, 2024), *Peperomia kauaiana* falls into the Critically Endangered (CR) category, indicating a high risk of extinction in the wild. Our formal evaluation can be summarised by the IUCN hierarchical alphanumeric numbering system of criteria and subcriteria, CR B1ab(iii)+2ab(iii), which reflects a severely limited extent of occurrence (EOO) of 12 km^2^, an area of occupancy (AOO) of less than 4 km^2^, a wild population of only three small sub-populations consisting of 1500–2100 mature plants and a continuing decline in the quality of habitat inferred.

The continued decline in quality of habitat for *P. kauaiana* is evidenced by severe habitat degradation from invasive non-native mammals, such as pigs (*Sus scrofa* L.), rats (*Rattus* spp.) and occasional goats (*Capra hircus*), along with introduced slugs, insects and disease. Other serious threats include hurricane-force winds, flash floods, landslides triggered after torrential rains and invasive non-native plants that displace naturally occurring ones within *P. kauaiana* habitat, especially *Sphaeropteris cooperi* (Hook. ex F. Muell.) R.M.Tryon (Cyatheaceae); *Melastoma septemnervium* Lour., *Miconia crenata* (Vahl.) Michelang. (Melastomataceae); *Psidium cattleyanum* Sabine, *Rhodomyrtus tomentosa* (Aiton) Hassk. (Myrtaceae); *Axonopus fissifolius* (Raddi) Kuhlm. (Poaceae); *Rubus rosifolius* Sm. (Rosaceae); and *Buddleja asiatica* Lour. (Scrophulariaceae).

##### ﻿Notes on two possibly extinct Hawaiian *Peperomia*

**Figure 7.**
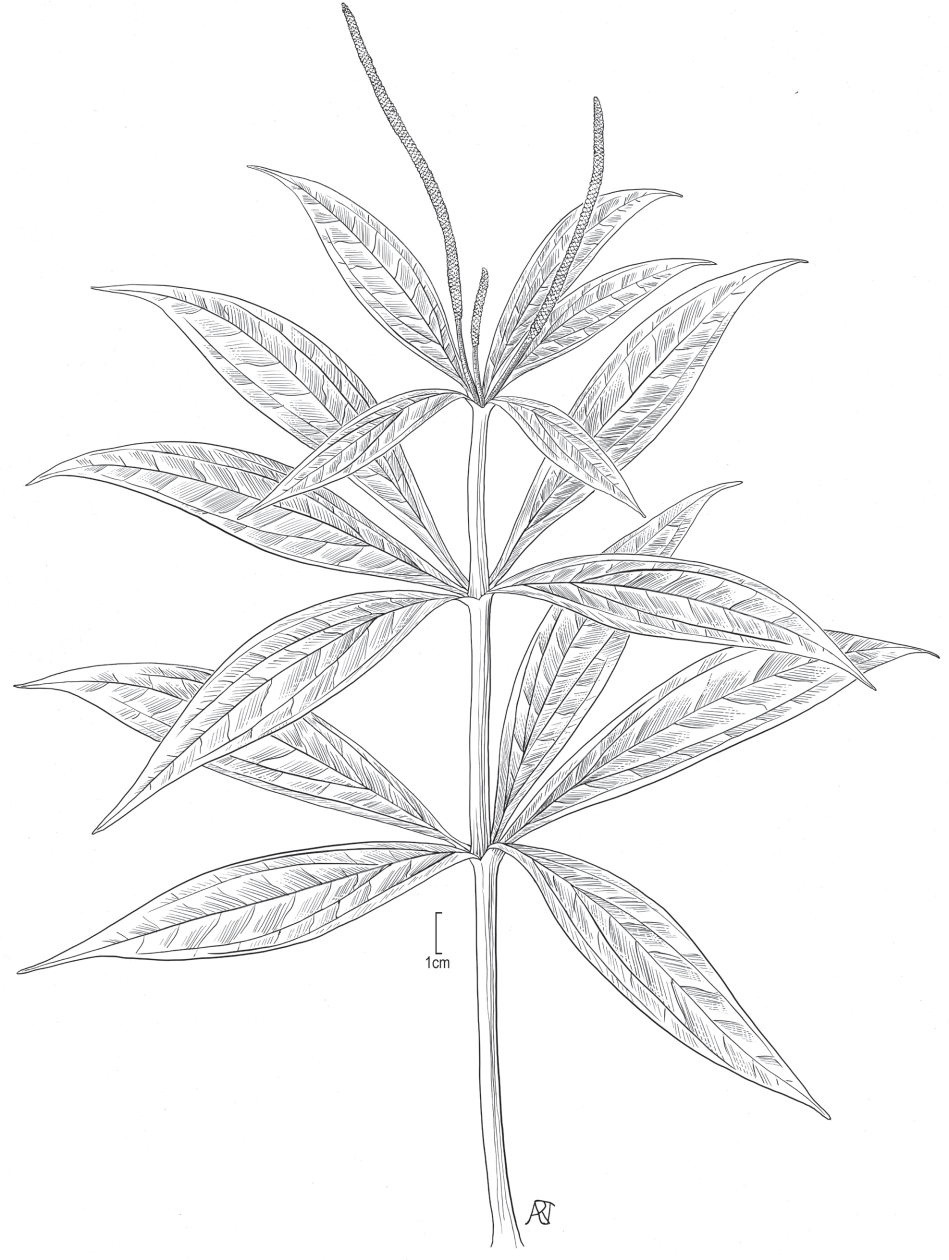
*Peperomia subpetiolata* Yunck. Habit showing stem, leaves and spikes drawn from: East Maui, Waikamoi Road, 29 Oct 1986, *R. Hobdyi 2636* (BISH) and leaf shape drawn from: East Maui, Waikamoi flume access road, 14 Mar 1984, *R. Hobdyi & G. Shishido 2017* (BISH). Illustration by Alice Tangerini.

Plant and animal endemics from isolated oceanic islands are more often critically endangered and highly susceptible to extinction, being particularly vulnerable and sensitive to invasive introductions of foreign plants and animals (Milberg and Tyrberg 1993; Kearns et al. 1998; Sakai et al. 2002; Wood 2012; Wood et al. 2019). The loss of suitable habitat on islands can be devastating to specialised species, especially considering their inherently limited land mass when compared to continental regions (Heinen et al. 2018). Currently, there are over 130 Hawaiian vascular plant taxa that are considered possibly extinct, including two Hawaiian *Peperomia* species, namely *P. degeneri* from Moloka‘i (Fig. 5) and *P. subpetiolata* Yunck. from Maui (Fig. 7) (Wood 2012; Wood et al. 2019). The following is a summary of where and when those two *Peperomia* species were observed and we encourage efforts be made to relocate them.

**Figure 8.**
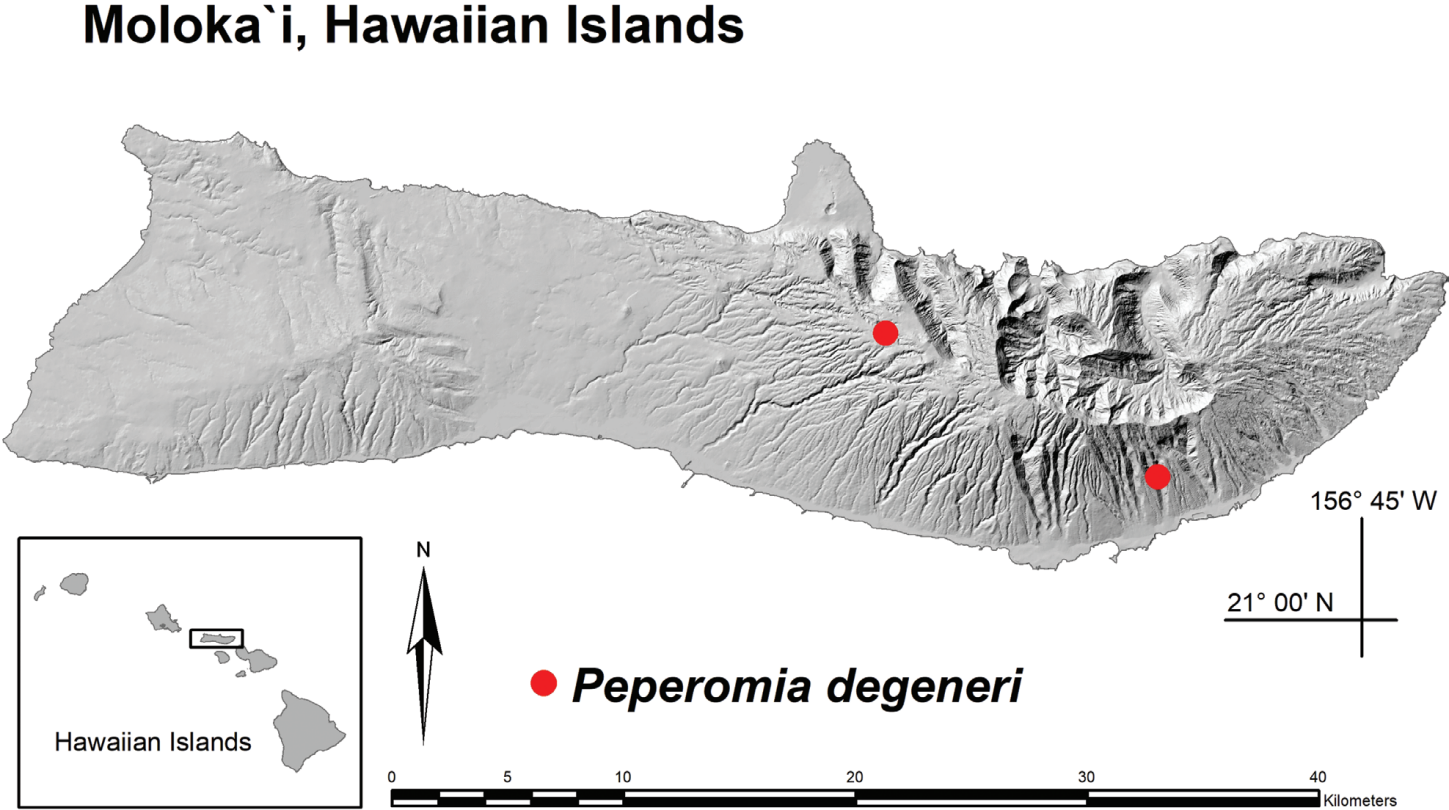
Distribution map with red dots indicating the two historical locations of *Peperomia degeneri*, Moloka‘i, Hawaiian Islands. Upper left dot is the Waihānau site and lower right dot is the Kalua‘aha site.

*Peperomia degeneri* (Fig. 5) has only been documented from two locations (Fig. 8). It was originally collected by O. Degener in 1928 on shaded cliffs along the east arm of Kalua‘aha Valley, Moloka‘i at an unrecorded elevation and was subsequently described by Yuncker in 1933. For many years, botanists studying the Hawaiian flora lost track of any wild populations and considered *P. degeneri* to be possibly extinct. Its re-discovery occurred in 2008, 80 years after Degener’s observation, when H. Oppenheimer made an herbarium collection around Waihānau Valley, Moloka‘i, at an elevation of 900 m. Unfortunately, when Oppenheimer returned to the site several years later, the colony of *P. degeneri* had perished, once again raising the possibility of its extinction.

It should be noted that, although Yuncker (1933) describes the spike of *Peperomia degeneri* as being up to 3 cm long, on careful examination of the isotype collections, we find that the spike (peduncle and fertile rachis) can be as long as 4.8 cm (see diagnosis and key to Hawaiian *Peperomia* with alternate leaves). It is also worth noting that *P. degeneri* has only been documented on vertical habitats, with Degener having found it on shaded cliffs and Oppenheimer on vertical stream banks.

Specimens of *Peperomia degeneri* examined. USA. Hawaiian Islands, Moloka‘i: • Kalua‘aha Valley, 12 Jul 1928 (fl., fr.), *Degener and Wiebke 3061* (isotypes: BISH, CAS, ILL, K, MASS, NY, RM, US) • Waihānau, 900 m alt., 25 Sep 2008, (fl., fr.), *Oppenheimer H90814* (BISH).

**Figure 9.**
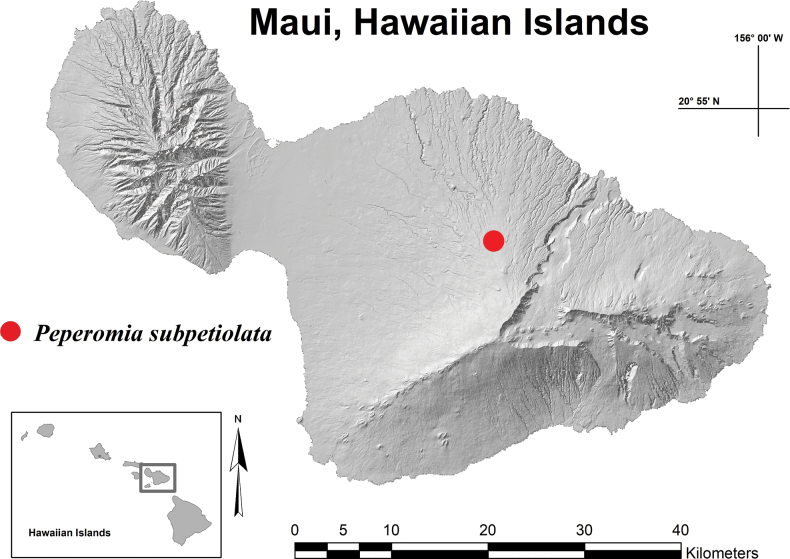
Distribution map with red dot indicating the only known historical location of *Peperomia subpetiolata* around the Kula Pipeline and Waikamoi Road region of East Maui, Hawaiian Islands.

*Peperomia subpetiolata* (Fig. 7) is a rare species on East Maui, known only from a small area near and along Olinda Road. This area is just west of the Makawao/Koolau forestry boundary line (Fig. 9). It is a highly distinctive Hawaiian species with leaves 5–8 per node, up to 20 cm long, linear-lanceolate to narrowly elliptic and a spike with a rachis 3.5–4 mm in diameter. Only 11 collections are known and were made in the general area of the Kula Pipeline and Waikamoi Road. No pure individuals of *P. subpetiolata* have been recorded since the late 1990s and only putative hybrids have been observed since then, most likely between *P. subpetiolata* × *P. cookiana* and/or *P. subpetiolata* × *P. hirtipetiola*.

*Peperomia kulensis* Yunck. originally described in 1933, based on a single specimen collected from below Kula pipeline on East Maui (*G. Munro 800* (BISH)) was treated as a synonym of *P. subpetiolata* by Wagner et al. (1990, 1999). The features that differed in the type and only collection were included within the description of *P. subpetiolata* by Wagner et al. (1990, 1999), primarily plant parts hirsute, leaves 9- to 11-nerved and the petiole more distinct, but narrowly winged. In 1995, the type was re-examined by Joel Lau and Wagner with the knowledge that *P. hirtipetiola* C. DC. also grew in those areas and concluded the type was most likely a hybrid and thus annotated the type as a putative hybrid *P. subpetiolata* × *P. hirtipetiola*. Since this putative hybrid is now removed from inclusion in *P. subpetiolata*, a new description is needed and is provided below.

**Description. *Herb***, terrestrial, stems greenish with reddish-purple splotches, erect to ascending from a very short repent base, not rooting at the nodes, stout, unbranched or 2- or 3-branched in upper part, ca. 60–150 cm long, 8–20 mm in diameter, internodes 3–4 cm long or up to 12 cm long in lower part of stem, glabrous. ***Leaves*** 5–8 per node, relatively thin, coriaceous, drying chartaceous, linear-lanceolate to narrowly elliptic, 12–20 cm long, 1.5–4 cm wide, palmately 5-nerved, veins impressed on upper surface, upper surface dark green, lower surface pale green, glabrous, apex long-acuminate, base attenuate, tapering to a short narrowly winged petiole, 0.2–1 cm long. ***Spikes*** 1 to 3, terminal and axillary, 8–12 cm long, peduncles ca. 2–3.5 cm long, 3.5–4 mm in diameter, glabrous, flowers densely congested; stamens ellipsoidal, 2; ovary turbinate; stigmas terminal, inconspicuously divided. ***Fruit*** subglobose, ca. 1 mm in diameter.

Specimens of *Peperomia subpetiolata* examined. USA. Hawaiian Islands, East Maui: • **Waikamoi**, Kula Pipeline, 5 Sep 1919, *Forbes 1283.M* (holotype: BISH) • *loc. cit.*, 1372 m alt., 11 Feb 1930, *St John 10299* (BISH) • *loc. cit.*, 1250 m alt., 14 Mar 1984, *Hobdy 2017* (BISH) • *loc. cit.*, 1250 m alt., 29 Oct 1986, *Hobdy 2635 & 2636* (BISH) • *loc. cit.*, 1250 m alt., 14 Mar 1984, *Wagner et al. 5675* (BISH).

## Supplementary Material

XML Treatment for Peperomia kauaiana

## ﻿Additional information

### Use of AI

No use of AI was reported.

### Author ORCIDs

Kenneth R. Wood https://orcid.org/0000-0001-6446-1154

Warren L. Wagner https://orcid.org/0000-0001-5012-8422

Susan Fawcett https://orcid.org/0000-0001-5021-1779

### Data availability

All of the data that support the findings of this study are available in the main text.
